# Breast cancer pathology image recognition based on convolutional neural network

**DOI:** 10.1371/journal.pone.0311728

**Published:** 2025-05-19

**Authors:** Weijian Fang, Shuyu Tang, Dongfang Yan, Xiangguang Dai, Wei Zhang, Jiang Xiong

**Affiliations:** 1 Chongqing Three Gorges University, Chongqing, China; 2 School of Computer Science and Engineering, School of Three Gorges Artificial Intelligence, and Key Laboratory of Intelligent Information Processing and Control, Chongqing Three Gorges University, Chongqing, China; Graphic Era Deemed to be University, INDIA

## Abstract

This study presents a convolutional neural network (CNN)-based method for the classification and recognition of breast cancer pathology images. It aims to solve the problems existing in traditional pathological tissue analysis methods, such as time-consuming and labour-intensive, and possible misdiagnosis or missed diagnosis. Using the idea of ensemble learning, the image is divided into four equal parts and sixteen equal parts for data augmentation. Then, using the Inception-ResNet V2 neural network model and transfer learning technology, features are extracted from pathological images, and a three-layer fully connected neural network is constructed for feature classification. In the recognition process of pathological image categories, the network first recognises each sub-image, and then sums and averages the recognition results of each sub-image to finally obtain the classification result. The experiment uses the BreaKHis dataset, which is a breast cancer pathological image classification dataset. It contains 7,909 images from 82 patients and covers benign and malignant lesion types. We randomly select 80% of the data as the training set and 20% as the test set and compare them with the Inception-ResNet V2, ResNet101, DenseNet169, MobileNetV3 and EfficientNetV2 models. Experimental results show that under the four magnifications of the BreaKHis dataset, the method used in this study achieves the highest accuracy rates of 99.75%, 98.31%, 98.51% and 96.69%, which are much higher than other models.

## Introduction

Breast cancer ranks among the prevalent forms of malignant tumors in women, posing a significant global health concern for women. The World Health Organisation (WHO) reports that breast cancer continues to be a pressing issue affecting millions of women annually, with a projected rise in new cases to reach 27 million by 2030 [1]. Timely identification and precise diagnosis of breast cancer play a pivotal role in enhancing treatment effectiveness and improving the chances of patient survival [2]. Currently, the main diagnostic methods for breast cancer are breast physical examination, breast ultrasonography, mammography, breast magnetic resonance imaging and breast biopsy [3]. The doctor will check for abnormalities by touching and looking at the breast. This includes checking for lumps, pain, skin changes, or nipple discharge. While breast ultrasound, mammography, and breast MRI are medical imaging tests that can assist in pinpointing the location of abnormalities, they are unable to provide a definitive diagnosis regarding the malignant nature of the identified area [4]. Breast biopsy is the most accurate method for confirming whether a region exhibits cancerous characteristics. However, the manual process is not only an arduous and time-consuming endeavor, but also necessitates the proficiency and expertise of a pathologist. This process demands an exceptionally high level of skill and knowledge from the pathologist. Moreover, the consensus rate among specialists generally hovers around 75%, which can be influenced by a multitude of factors [5]. This may result in varying diagnostic outcomes for the same specimen among different pathologists. The adoption of computer-aided diagnostic (CAD) systems can mitigate this variability, while also easing the pathologists’ workload, enhancing work efficiency, reducing testing costs, and ensuring a high level of accuracy [6].

In recent years, the rapid advancement of deep learning technology has significantly contributed to the field of medicine, where industrial intelligence technology now holds a pivotal role [7]. Many scholars have achieved fruitful research results through this technique [8–14], advancing the development of deep learning technology in the field of medical image diagnosis. Deep learning technology, characterized by multi-layer neural network learning algorithms, has the capability to acquire features via intricate deep nonlinear network structures. It achieves this by amalgamating low-level features to construct more abstract deep representations, often referred to as attributes or features within categories. Deep learning eliminates the need for manual feature design or extraction, enabling complex function approximation and the creation of distributed representations of input data to learn essential dataset features. Notably, convolutional neural networks and their variations have shown outstanding performance in image processing [15], thereby providing a solid foundation for the application of deep learning in breast cancer pathology image recognition [16]. Transfer learning [17], a significant subfield within deep learning, has gained considerable popularity and holds great promise in machine learning due to its extensive range of applications [18]. In the context of medical image recognition, its utilization can enhance model performance when dealing with limited sample data, as it leverages knowledge from the source domain [19]. By fine-tuning a pre-trained model, the knowledge acquired from the source domain data can expedite the convergence process of the target task, thereby accelerating model training. Employing the pre-trained model as initial parameters or keeping the underlying feature extractor fixed can also lead to a reduction in the model’s parameter count for the target task, subsequently lowering the risk of overfitting [20].

Automatically classifying breast cancer pathology images presents significant challenges. Firstly, the inherent traits of pathological images, such as subtle variations between images, cell overlap, and uneven color distribution, pose substantial obstacles to image classification. Secondly, the limited availability of large publicly labeled datasets presents challenges for algorithmic research.

### Related work

As computer technology continues to advance, numerous scholars have explored the application of computer-aided diagnostic techniques for breast cancer pathology image recognition, yielding a range of notable outcomes. Currently, research in breast cancer recognition primarily centers on the following two aspects:

(1) Breast cancer pathology image classification through manual feature extraction and traditional machine learning algorithms. Kowal *et al*. [21] applied various kernel segmentation algorithms and attained recognition rates ranging from 96% to 100% on a dataset comprising 500 breast cancer pathological images. Zhang *et al*. [22] introduced a method that utilizes specially crafted features in a single-class kernel principal component analysis. This method was employed to classify 361 pathological breast cancer images, achieving a recognition accuracy of 92%. Belsare *et al*. [23] used statistical texture features to train k-nearest neighbour (K-NN) and support vector machine (SVM) classifiers to achieve 70% to 100% accuracy on a private breast histology dataset with 40x magnification. Wang *et al*. [24] classified 68 breast cancer pathology images with 96.19% accuracy using a support vector machine algorithm. It is evident that the majority of the aforementioned algorithms designed for classifying breast cancer pathology images have been evaluated on relatively small datasets. This can lead to a lack of standardized performance benchmarks for comparing these algorithms across different studies. Moreover, there is also the problem of process complexity in manual design and feature extraction.

(2) Image classification for breast cancer pathology using deep learning techniques. In recent years, Convolutional Neural Networks (CNNs), a pivotal deep learning technique, have demonstrated remarkable accomplishments in the domain of image recognition. On the one hand, deep learning allows the model to extract features directly from the input image, avoiding the complexity and limitations of manually designing and extracting features in the traditional algorithms, and saving a lot of human and material resources. On the other hand, Convolutional Neural Networks (CNNs) have found extensive applications in various domains including natural language processing, object recognition, image classification, and medical image analysis [25,26]. Araújo *et al*. [27] employed a convolutional neural network (CNN) for the classification of breast cancer pathology images into cancerous and non-cancerous categories, achieving an impressive recognition rate of up to 88.3%. The highest overall accuracy of 77.8% was achieved when further classified into four categories: normal tissue, benign lesions, carcinoma in situ and invasive carcinoma. Spanhol *et al*. [28] employed a transfer learning methodology to extract deep features from a collection of breast cancer histopathology images. They utilized the pre-trained BVLC CaffeNet architecture’s weights and inputted these features into a classifier, achieving an accuracy ranging from 83.6% to 84.8%. Bayramoglu *et al*. [29] introduced an method that is independent of magnification factors for the classification of breast cancer histopathological images, utilizing the BreaKHis dataset. This method simultaneously categorizes pathology images as benign or malignant while also determining the magnification level. Their experimental findings yielded an accuracy of 84.3% in the classification of benign and malignant cases. Nonetheless, there remains room for improvement in the recognition rates of these methodologies in order to fully harness the potential of Computer-Aided Diagnosis (CAD) systems and better align with their clinical applications.

This study proposes a method based on Convolutional Neural Network (CNN) for the precise classification and recognition of breast cancer pathological images. This method utilizes the concept of ensemble learning for data augmentation, and adopts the Inception-ResNet V2 neural network model combined with transfer learning technology to extract image features. On this basis, a neural network with a three-layer fully connected structure is constructed to achieve accurate classification of features. In the recognition process of pathological image categories, the network first identifies each sub-image, and then performs summation and averaging on the recognition results of each sub-image to finally obtain the classification result. In the experiment, the BreaKHis dataset was selected, and a comprehensive comparison was made with multiple deep learning models, which proved the effectiveness of our method. The most significant contributions of the article are presented as follows:

(1) Improving the traditional method by using CNNs for automatic feature extraction, enhancing diagnostic efficiency and accuracy.
(2) Adopting the Inception-ResNet V2 model and migration learning to help the model converge faster and adapt better to the classification task.
(3) Applying data enhancement techniques to expand the dataset, increase sample diversity, reduce overfitting risk, and improve the model’s generalization ability.

## Materials and methods

### Dataset

In this study, we employ the publicly accessible BreaKHis dataset (Breast Cancer Histology Image Database) [30] for breast cancer histopathological image analysis, from the Federal University of Paraná in their paper, which has been widely used in research on breast cancer image recognition and analysis. The dataset contains a total of 7,909 annotated histopathological images of breast cancer from 82 patients, with 2,480 images of benign tumours and 5,429 images of malignant tumours. Each breast tumour section was stained with haematoxylin and eosin (HE staining). The dataset consisted of eight benign and malignant tumour types. The four benign tumour types included: adenomas, fibroadenomas, lobular tumours and tubular adenomas. The four categories of malignant tumors comprise ductal carcinoma, lobular carcinoma, mucinous carcinoma, and papillary carcinoma. These images were acquired using four magnifications (40×, 100×, 200×, and 400×), all sized as 700×460 R, G, and B three-channel images. The distribution of samples at each magnification is shown in Table 1.

**Table 1:** Number of benign and malignant pathology images at different magnification factors. From 82 patients totaling 7909 pathologic images, 24 benign patients totaling 2480 pathologic images and 58 malignant patients totaling 5429 pathologic images.

Magnification factor	Benign	Malignant	Total
40×	625	1370	1995
100×	644	1437	2081
200×	623	1390	2013
400×	588	1232	1820
Total	2480	5429	7909
Number of patients	24	58	82

**Fig 1:**
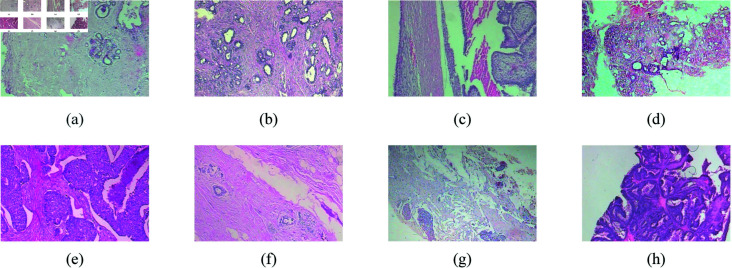
Sample of breast cancer histopathology image at 40× magnification. a d are pathologic pictures of benign and e h are malignant, and their names are (a) adenosis, (b) fibroadenoma, (c) phyllodes_tumor, (d) tubular_adenoma, (e) ductal_carcinoma, (f) lobular_carcinoma, (g) mucinous_carcinoma, (h) papillary_carcinoma.

In this study, our focus is solely on the binary classification task, distinguishing between benign and malignant breast cancer pathology images at four different magnification levels, and in Fig 1, we present sample images depicting four instances of benign and four instances of malignant breast cancer pathology at a 40× magnification level.

### Data augmentation methods

**Fig 2:**
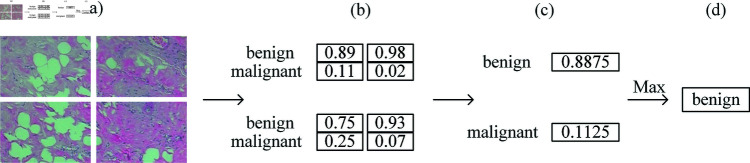
The recognition process after four-equal segmentation of pathology images. (a) Input the pathology images that are divided into four equal parts, (b) calculate the probability of belonging to benign and malignant for each sub-image, (c) the probabilities of benign and malignant for each sub-image are summed to give the probability of a picture being benign and malignant, and (d) compare the probabilities of benign and malignant, and select the maximum probability as the classification result.

Given that the BreaKHis dataset comprises only 7909 annotated pathological breast cancer images, with 1995, 2081, 2013, and 1820 images at magnifications of 40×, 100×, 200×, and 400× respectively, such a sample size is notably inadequate for training deep neural networks. Due to the insufficient amount of data, it may lead to model overfitting and reduced generalisation ability. Nonetheless, obtaining a substantial volume of medical images, particularly those expertly labeled by healthcare professionals, is a daunting and expensive endeavor. Moreover, image enhancement techniques have long been a focal point of numerous scholars, offering an effective means to bolster model performance [31–34]. Therefore, data augmentation becomes essential to augment the pool of training samples, thereby enhancing the training dataset’s size, as well as improving the model’s robustness and generalization capabilities. In this paper, we use two data enhancement methods. Firstly, we use traditional data enhancement techniques including methods like horizontal and vertical flipping, scaling, random rotation, color transformation, and adding noise. Another data enhancement method is to use the idea of ensemble learning [35,36] to crop the graph original pathology into equal sized 4 and 16 parts respectively, and when predicting the pathology image categories, the average fusion algorithm is used [37], which combines the segmented pathology image classification results to get the prediction results of pathology pictures, As depicted in Fig 2.

For each category j, the average probability value is calculated:

$$Average\;probility\;for\;class\;\;j\;=\frac{1}{K}\sum_{k=1}^{K}y_{i}^{k}[j],$$ (1)

where $y_{i}^{k}$ denotes the probability value that the kth model predicts category j for the ith sample. The final prediction results in the category with the highest probability value, i.e:

$$Final\;predicted\;class\;for\;sample\;\;i\;=argmax\left(\frac{1}{K}\sum_{k=1}^{K}y_{i}^{k}[j]\right)$$ (2)

**Fig 3:**
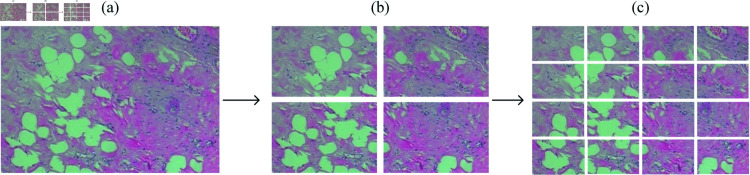
The process of image segmentation. (a) Original image, (b) dividing the image into four equal parts, (c) dividing the image into sixteen equal parts.

As depicted in Fig 3, following segmentation, the training data volume is expanded to four and sixteen times the original dataset size, respectively. Each sub-image, resulting from the segmentation, is assigned the same class label as the original image.

### Inception-ResNet V2

**Fig 4:**
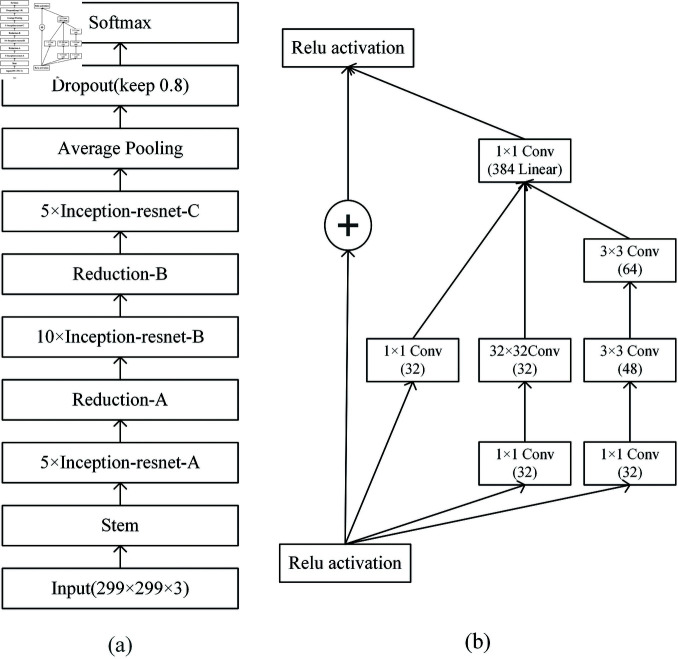
Inception-ResNet V2 network architecture. The figure (a) shows the complete network structure of the Inception-ResNet V2 neural network model and (b) shows detailed structure of Inception-Resnet-A.

Inception-ResNet V2 [38], as introduced by Google in 2016, is a deep convolutional neural network architecture depicted in Fig 4. It amalgamates features from both the Inception and ResNet model families. The architecture performed well in the 2016 ILSVRC image classification benchmark, achieving an excellent Top-5 error rate of about 3.08%.

The Inception module was originally introduced by Christian Szegedy *et al*. in 2014, is a new deep learning framework also known as GoogleNet.The Inception module captures image features at different scales and abstraction levels by performing various convolutional and pooling operations (e.g., 1×1, 3×3, or 5×5) to obtain better image feature attributes. Such a module comprises several parallel convolutional and pooling layers, with each parallel branch utilizing a convolutional kernel of varying sizes. This design enables the network to concurrently learn both local and global features, thereby enhancing the efficiency of feature extraction.

Nevertheless, with the expansion of network depth, there is a corresponding increase in parameters and computations, potentially giving rise to the problem of vanishing gradients. To overcome this challenge, based on the Inception module, Inception-ResNet V2 introduces ResNet37 residual connection in the Inception module. This connection facilitates the direct transfer of information from shallower layers to deeper layers, effectively addressing the issue of gradient vanishing in deep networks. The design of the residual network makes it easier to pass gradient information during backpropagation, which speeds up convergence. By integrating the Inception module with ResNet’s residual connections, Inception-ResNet V2 achieved outstanding performance in image classification tasks, establishing itself as one of the prominent deep learning architectures of its era. This combination effectively merges the strengths of both architectures, improving the performance and efficiency of the network.

### Transfer learning

Transfer learning [39–41] is a method that draws on existing machine learning models to solve practical problems. Its core idea is to transfer the knowledge and skills learned in one domain to another related domain, especially when the target domain lacks sufficient labeled data. Typically, a pre-trained model on a large amount of data is used as a starting point to initialize the model parameters of the target task. By fine-tuning this pre-trained model, its parameters can be adjusted to adapt to the specific data of the target task.

In this study, transfer learning technology is adopted to improve the accuracy of the classification of breast cancer pathological images. The pre-trained Inception-ResNet V2 weights on the ImageNet dataset [42] are used as the initialization weights to initialize the model parameters of the target task, which can leverage the existing knowledge to accelerate the learning process of the model.

### Feature extraction and image classification

In the realm of deep learning, numerous outstanding convolutional neural network (CNN) models have been introduced, including LeNet, VGG, AlexNet, and ResNet. These models have achieved remarkable results on image recognition tasks. Constructing new models based on these mature models often gives better results and is more convenient and faster. For example, feature extraction of breast images was performed based on the AlexNet model [43], the model is relatively simple, which limits its classification accuracy. In this study, we opted for the Inception-ResNet V2 neural network model to construct the model for classifying pathological images.

**Fig 5:**
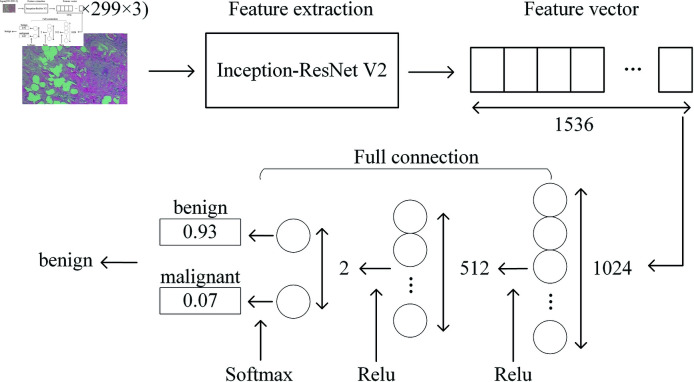
Pathological image recognition process. The input image is first compressed to 299×299×3 and fed into the Inception-ResNet V2 model for feature extraction, and then the pathology image is classified using a three-layer fully connected neural network.

The model’s architecture, illustrated in Fig 5, comprises two main components: a feature extraction process and a classification process. In the feature extraction process, the image was resized to meet the Inception-ResNet V2 model’s requirement of a 299×299 input image size, and we employed the Inception-ResNet V2 neural network model to perform feature extraction. In this process, the final fully connected layer of the Inception-ResNet V2 neural network model is removed.

The classification process involves the inclusion of three additional fully connected neural network layers, with the last three layers having 1024, 512, and 2 nodes, respectively. The first two layers of fully connected neural network use ReLU [44] as the activation function, defined as follows:

$$ReLU(x)=\left\{ \begin{array}{cc} x & \;if\;x>0\\ 0 & \;if\;x\leq 0 \end{array}\right.$$ (3)

The last layer is classified using SoftMax as the activation function, defined as follows:

$$\sigma (\tilde{z}{)}_{i}=\frac{e^{z_{i}}}{\sum_{j=1}^{K}e^{z_{j}}}$$ (4)

The model uses cross entropy as the loss function, defined as follows:

$$Loss=-\frac{1}{S}\sum_{i=1}^{S}\sum_{j=1}^{C}y_{ij}•log({y_{ij}}^{p})$$ (5)

Given the limited dataset, this paper focuses on training only the parameters of the three fully connected layers following feature extraction. To acquire the parameters of the Inception-ResNet V2 neural network model during the feature extraction stage, a transfer learning approach is employed. Specifically, the Inception-ResNet V2 neural network model parameters pre-trained on the ImageNet dataset are used as initialised weights for the model in the feature extraction phase. This approach offers the benefit of expediting the model’s convergence on the target task by harnessing the general features acquired from extensive datasets. The use of fine-tuning leads to changes in the model parameters. The feature vectors of the image need to be recalculated for each training, which can result in a huge computational task and training time. To alleviate the computational load, this paper employs a fixed weights strategy. This approach significantly decreases the computational requirements during the feature extraction phase, expedites the training process, and mitigates the risk of overfitting to the learned generic features. This means that in the feature extraction phase, the weights of the model remain unchanged and are not involved in the training process. Parameter tuning and training are performed only in the subsequent fully connected layer.

### Experiments

In this paper, all experiments were done in the same test environment. The test environment is based on a computer with an Intel I5-13600kf processor, an NVIDIA RTX 2060 Super 8GB video memory GPU and 32GB of RAM. The test operating system was Windows 11 and was programmed using the TensorFlow architecture in the Python language.

In this experiment, the dataset is divided into a training set and a test set, with a split ratio of 80% and 20% respectively, and the data distribution is shown in Table 2.

**Table 2:** Training and test set distribution. The distribution of benign and malignant tumor image data in the training and test sets at different magnifications.

Magnification Factors	Category	Train	Test
40×	bengin	500	125
	maligant	1096	274
100×	bengin	516	128
	maligant	1150	287
200×	bengin	499	124
	maligant	1112	278
400×	bengin	417	117
	maligant	986	246

The training set is employed for training the model’s hyperparameters, while the test set serves as the means to assess the model’s performance. We employed the Adaptive Moment Estimation (Adam) gradient optimization algorithm [45] to update the weights and biases. The initial learning rate was set to 0.001, and we considered both 0.9 and 0.999 as the momentum factors. Each training iteration consisted of 32 batches. The classification of medical images is typically evaluated from two perspectives: patient-level and image-level. As the BreakHis dataset contains only 82 patients, in this study, we specifically employ image-level assessment to determine the model’s recognition accuracy. The evaluation metrics include Accuracy, Precision, Recall, F1 score and MCC [46], defined as follows:

$$Accuracy=\frac{TP+TN}{TP+TN+FP+FN}$$ (6)

$$Precision=\frac{TP}{TP+FP}$$ (7)

$$Recall=\frac{TP}{TP+FN}$$ (8)

$$F1\;score=\frac{2\times Precision\times Recall}{Precision+Recall}$$ (9)

$$MCC=\frac{TP\times TN-FP\times FN}{\sqrt{\left(TP+FP)(TP+FN)(TN+FP)(TN+FN\right)}}$$ (10)

Among them, TP (True Positive) represents the number of samples correctly predicted as positive categories. In this study, the main focus is on malignant breast cancer pathological images. Therefore, malignancy is set as true positive. FN (False Negative) represents the number of samples incorrectly predicted as negative categories. FP (False Positive) represents the samples incorrectly predicted as positive categories. TN (True Negative) represents the samples correctly predicted as negative categories. Together, they form a confusion matrix used to evaluate the performance of binary classification models.

## Results

### Ablation experiment

An ablation study was conducted employing diverse model training strategies to ascertain the model’s performance across a spectrum of experimental configurations. The configurations encompassed data augmentation, the utilization of pre-trained models, conventional data augmentation techniques, quarter-image data enhancement, and sixteenth-image data intensification, as delineated in Table 3.

**Table 3:** The experimental results of this study model under different training strategies. Under the evaluation metrics of accuracy, precision, recall and F1_Score, the effect is best when using an image divided into sixteen equal parts combined with a pre-training strategy.

Indicator	Transfer Learning	Data Processing	Magnification Factors			
			40×	100×	200×	400×
Accuracy	None	None	89.47	79.76	80.60	85.79
	ImageNet	None	94.99	92.29	93.28	89.81
	ImageNet	Routine Data Augmentation	95.99	95.42	94.03	92.56
	ImageNet	Quarter Division	98.25	96.39	97.26	94.22
	ImageNet	Sixteenth Division	99.75	98.31	98.51	96.69
Precision	None	None	99.27	75.89	74.91	90.52
	ImageNet	None	95.68	94.12	94.04	91.30
	ImageNet	Routine Data Augmentation	96.40	96.85	94.10	92.61
	ImageNet	Quarter Division	98.54	98.96	98.92	97.97
	ImageNet	Sixteenth Division	99.64	99.30	99.64	98.37
Recall	None	None	87.18	93.04	95.81	87.87
	ImageNet	None	97.08	94.77	96.40	93.90
	ImageNet	Routine Data Augmentation	97.81	96.52	97.48	96.75
	ImageNet	Quarter Division	98.90	95.95	97.17	93.77
	ImageNet	Sixteenth Division	100.00	98.28	98.23	96.80
F1_Score	None	None	92.83	83.59	84.08	89.17
	ImageNet	None	96.38	94.44	95.20	92.59
	ImageNet	Routine Data Augmentation	97.10	96.68	95.76	94.63
	ImageNet	Quarter Division	98.72	97.43	98.04	95.83
	ImageNet	Sixteenth Division	99.82	98.79	98.93	97.58
MCC	None	None	75.56	59.95	63.21	68.60
	ImageNet	None	88.27	81.85	84.08	76.37
	ImageNet	Routine Data Augmentation	90.62	89.29	85.84	82.75
	ImageNet	Quarter Division	95.93	91.48	93.55	86.64
	ImageNet	Sixteenth Division	99.42	96.03	96.50	92.39

**Fig 6:**
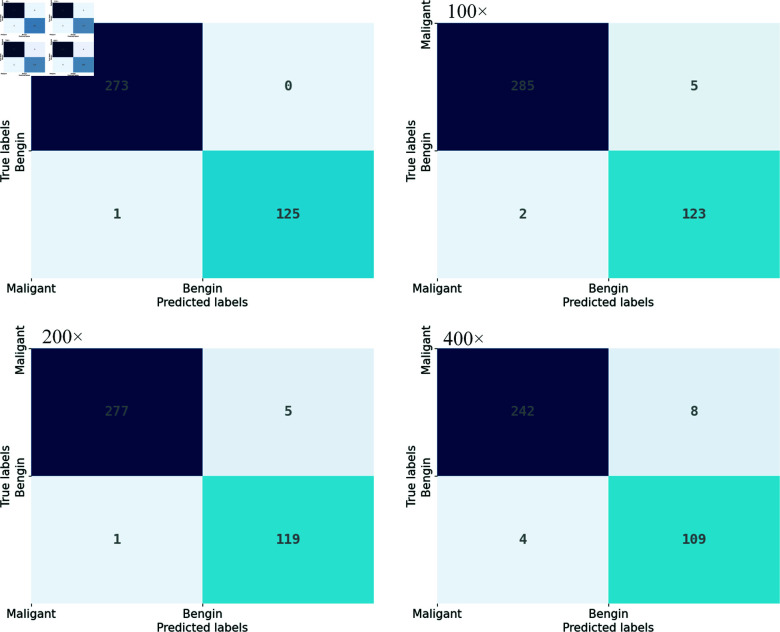
Confusion matrix. Using the 16-segment image augmentation strategy, the confusion matrix for 40× magnification is in the top left, 100× in the top right, 200× in the bottom left, and 400× in the bottom right.

Table 2 demonstrates that the incorporation of transfer learning and data augmentation techniques has significantly improved the model’s accuracy, precision, recall, and F1 score. By employing a 16-segment image enhancement strategy at magnifications of 40×, 100×, 200×, and 400×, the model achieved accuracy rates of 99.75%, 98.31%, 98.51%, and 96.69% respectively, precision rates of 99.64%, 99.30%, 99.64%, and 98.37% respectively, recall rates of 100.00%, 98.28%, 98.23%, and 96.80% respectively, and F1 scores of 99.82%, 98.79%, 98.93%, and 97.58% respectively. As illustrated in the confusion matrix shown in Fig 6, under the 16-segment strategy, at a 40× magnification, only one sample was incorrectly predicted as malignant; at a 100× magnification, two samples were incorrectly predicted as malignant; at a 200× magnification, five samples were incorrectly predicted as malignant; and at a 400× magnification, eight samples were incorrectly predicted as malignant. The experimental results indicate that the methods used have significantly enhanced the performance in identifying breast cancer pathology images.

### Comparison with other deep learning models

To comprehensively assess the model introduced in this study, we selected the best experimental results under each different amplification factor and compared them with ResNet101 [47], DenseNet169 [48], MobileNetV3 Large [49], and Inception-ResNet V2 [38] models for a detailed comparison. These model approaches utilize the same evaluation criteria as those employed in this paper, with the image-level recognition rate serving as the primary evaluation metric, as illustrated in Table 4. The comparative results indicate that under all four different magnifications, the Accuracy, Precision, Recall, F1_Score, and MCC of the method introduced in this study are consistently superior to other classification methods. This demonstrates the effectiveness of the training strategy adopted in this paper and the robustness of the deep learning model.

**Table 4:** Comparison with other deep learning models. Compared with other models, under the evaluation metrics of accuracy, precision, recall, F1_Score, and MCC, the effect is best when using an image divided into sixteen equal parts combined with a pre-training strategy.

Indicator	Methodologies	Magnification Factors			
		40×	100×	200×	400×
Accuracy	ResNet101	37.59	50.12	77.11	80.99
	DenseNet169	75.94	75.18	78.11	85.68
	MobileNetV3	83.96	82.41	82.34	86.23
	Inception-ResNet V2	87.97	78.80	78.61	84.57
	ours	99.75	98.31	98.51	96.69
Precision	ResNet101	9.12	33.10	99.64	96.75
	DenseNet169	99.64	66.55	100.00	84.96
	MobileNetV3	87.50	86.39	88.19	93.36
	Inception-ResNet V2	85.99	95.02	98.48	92.04
	ours	99.64	99.30	99.64	98.37
Recall	ResNet101	100.00	86.36	75.27	79.60
	DenseNet169	74.19	96.47	75.96	93.30
	MobileNetV3	89.42	88.50	85.97	85.77
	Inception-ResNet V2	98.54	73.17	70.14	84.55
	ours	100.00	98.28	98.23	96.80
F1_Score	ResNet101	16.72	47.86	85.76	87.34
	DenseNet169	85.05	78.76	86.34	88.94
	MobileNetV3	88.45	87.44	87.07	89.41
	Inception-ResNet V2	91.84	82.68	81.93	88.14
	ours	99.82	98.79	98.93	97.58
MCC	ResNet101	17.46	22.37	43.58	54.71
	DenseNet169	40.95	56.48	46.96	69.36
	MobileNetV3	62.27	58.18	59.29	70.34
	Inception-ResNet V2	71.75	59.78	62.57	66.69
	ours	99.42	96.03	96.50	92.39

## Conclusion

This study proposes a method for the classification and recognition of breast cancer pathological images based on the Convolutional Neural Network. By using the Inception − ResNet V2 neural network for feature extraction and a three-layer fully connected neural network for image classification, combined with transfer learning technology and data enhancement methods based on ensemble learning.

Experimental results show that the data enhancement strategy of dividing the image into sixteen equal parts and the pre-training strategy significantly improve the accuracy, F1 score, and MCC of the model, and the recognition accuracy at four different magnification factors is higher than that of other deep learning models, proving the effectiveness of the training strategy and the robustness of the deep learning model. This study provides an effective method for the automated classification of breast cancer pathological images, which helps to improve the diagnostic efficiency and accuracy, and provides a valuable reference for clinical applications.

However, this study has some limitations, such as the dataset used may be limited in terms of sample representativeness and diversity, the model performance may be affected by factors such as image quality, staining changes, and the complexity of pathological features, and it only focuses on binary classification, and further research is needed for more detailed classification or multi-class tasks. Applying the model to actual clinical diagnosis may face challenges, such as the need for further verification and integration with the existing workflow. Future research can further explore methods to optimize the model performance and the way to apply this method to actual clinical diagnosis.

## Supporting information

S1 File (PDF)
